# Does Blast Exposure to the Torso Cause a Blood Surge to the Brain?

**DOI:** 10.3389/fbioe.2020.573647

**Published:** 2020-12-17

**Authors:** Jose E. Rubio, Maciej Skotak, Eren Alay, Aravind Sundaramurthy, Dhananjay Radhakrishnan Subramaniam, Vivek Bhaskar Kote, Stewart Yeoh, Kenneth Monson, Namas Chandra, Ginu Unnikrishnan, Jaques Reifman

**Affiliations:** ^1^Department of Defense Biotechnology High Performance Computing Software Applications Institute, Telemedicine and Advanced Technology Research Center, United States Army Medical Research and Development Command, Fort Detrick, MD, United States; ^2^The Henry M. Jackson Foundation for the Advancement of Military Medicine, Inc., Bethesda, MD, United States; ^3^Department of Biomedical Engineering, Center for Injury Biomechanics, Materials, and Medicine, New Jersey Institute of Technology, Newark, NJ, United States; ^4^Blast Induced Neurotrauma Division, Walter Reed Army Institute of Research, Silver Spring, MD, United States; ^5^Department of Biomedical Engineering, College of Engineering, The University of Utah, Salt Lake City, UT, United States; ^6^Department of Mechanical Engineering, College of Engineering, The University of Utah, Salt Lake City, UT, United States

**Keywords:** traumatic brain injury, indirect mechanism, blast overpressure, fluid-structure interaction, shock tube

## Abstract

The interaction of explosion-induced blast waves with the torso is suspected to contribute to brain injury. In this indirect mechanism, the wave-torso interaction is assumed to generate a blood surge, which ultimately reaches and damages the brain. However, this hypothesis has not been comprehensively and systematically investigated, and the potential role, if any, of the indirect mechanism in causing brain injury remains unclear. In this interdisciplinary study, we performed experiments and developed mathematical models to address this knowledge gap. First, we conducted blast-wave exposures of Sprague-Dawley rats in a shock tube at incident overpressures of 70 and 130 kPa, where we measured carotid-artery and brain pressures while limiting exposure to the torso. Then, we developed three-dimensional (3-D) fluid-structure interaction (FSI) models of the neck and cerebral vasculature and, using the measured carotid-artery pressures, performed simulations to predict mass flow rates and wall shear stresses in the cerebral vasculature. Finally, we developed a 3-D finite element (FE) model of the brain and used the FSI-computed vasculature pressures to drive the FE model to quantify the blast-exposure effects in the brain tissue. The measurements from the torso-only exposure experiments revealed marginal increases in the peak carotid-artery overpressures (from 13.1 to 28.9 kPa). Yet, relative to the blast-free, normotensive condition, the FSI simulations for the blast exposures predicted increases in the peak mass flow rate of up to 255% at the base of the brain and increases in the wall shear stress of up to 289% on the cerebral vasculature. In contrast, our simulations suggest that the effect of the indirect mechanism on the brain-tissue-strain response is negligible (<1%). In summary, our analyses show that the indirect mechanism causes a sudden and abundant stream of blood to rapidly propagate from the torso through the neck to the cerebral vasculature. This blood surge causes a considerable increase in the wall shear stresses in the brain vasculature network, which may lead to functional and structural effects on the cerebral veins and arteries, ultimately leading to vascular pathology. In contrast, our findings do not support the notion of strain-induced brain-tissue damage due to the indirect mechanism.

## Introduction

The interaction of explosion-induced blast waves with the human body is suspected to cause traumatic brain injury (TBI) by two prevailing mechanisms. One hypothesis is the *direct* mechanism, where the blast wave directly interacts with the head resulting in injury to the brain by one or a combination of effects, such as skull flexure (Bolander et al., 2011), head acceleration (Goldstein et al., 2012), cavitation (Goeller et al., 2012; Salzar et al., 2017), and pressure propagation through the skull and within the brain (Taylor and Ford, 2009; Chavko et al., 2011; Leonardi et al., 2011). The second hypothesis is the *indirect* mechanism, where the blast wave interacts with the body, compresses the abdomen and chest, and transfers its kinetic energy to the body organs, including the brain and the blood as a fluid medium (Cernak, 2010, 2015).

The seminal experimental animal studies by Clemedson and Criborn (1955); Clemedson and Pettersson (1956), and Clemedson (1956) helped establish the underlying theory for the indirect mechanism characterized by the wave-body interaction. Their work was among the first to suggest that a fraction of the shock-wave energy is absorbed and propagated through the body, including the central nervous system, as a tissue-transmitted wave. In addition, other studies investigated the propagation of ballistic-induced pressure waves through the body of pigs (Suneson et al., 1990a, b), suggesting that the kinetic-energy transfer from the pressure wave to the tissues could potentially damage the central nervous system. While these studies provided valuable insights on how the pressure wave could propagate through the body and reach the brain, the potential role of the indirect mechanism in causing blast-induced injury remains inconclusive, mainly due to the lack of careful experimental studies that appropriately isolate the indirect mechanism from competing possibilities and comprehensive computational studies that systematically investigate its potential effects on the brain vasculature and surrounding tissues.

While a few experimental studies have attempted to investigate the effects of the indirect mechanism on brain tissue, it is unclear whether the study experimental design allowed for proper isolation of the indirect mechanism from other potential causes of brain injury. For example, Cernak (2010) and Koliatsos et al. (2011) conducted shock-tube experiments by exposing mice to a blast wave while separately shielding their head or their torso. However, because the entire animal was placed inside the shock tube and information regarding the incident pressure under the shielded portion of the animal’s body was not provided, it is uncertain whether such an experimental setup allowed for proper isolation of the head or the torso from the incident blast wave. Similarly, in an attempt to characterize the effect of the indirect mechanism in rats, other studies have used blast-wave simulators that targeted shock waves to the thorax of the animal (Assari et al., 2013; Simard et al., 2014). However, because in this experimental setup the animals are positioned at the open end of the blast-wave simulator, they could have been exposed to jet-wind effects, which are not representative of a blast-overpressure (BOP) waveform exposure observed in open-field explosions (Cernak, 2015; Needham et al., 2015), that could have confounded the resulting observations.

The indirect-mechanism hypothesis suggests that during the interaction with the body surface, the shock wave compresses the abdomen and chest, and transfers its kinetic energy to the body’s internal structures, including the blood as a fluid medium (Cernak, 2010; 2015). In our study, we investigate whether the kinetic energy transferred to the brain (i.e., a body internal structure) through the blood (i.e., the fluid medium) could potentially damage the brain vessels and tissues. Surprisingly, despite extensive experimental evidence supporting varying degrees of cerebrovascular pathology in animals exposed to whole-body blast (Gama Sosa et al., 2013, 2014, 2019; Kuriakose et al., 2018, 2019; Logsdon et al., 2018; Heyburn et al., 2019), the existence of such a blood surge has not been thoroughly investigated, possibly because of the challenges in measuring hemodynamic parameters, such as mass flow rate and flow-velocity fields, in the cerebral vasculature during a blast exposure. As an alternative, and complementary to animal experimentation, high-fidelity, fluid-structure interaction (FSI) computational models allow for the characterization of such hemodynamic parameters and the estimation of biomechanical responses (e.g., the wall shear stress) in the cerebral vasculature, which could help elucidate whether blast exposure targeting only the torso can generate a noticeable blood surge to the brain.

To overcome these limitations and characterize the potential role of the indirect mechanism in causing blast-induced TBI, here we first conducted shock-tube experiments in rats, where we exposed only the torso of the animal to the incident blast wave by positioning the head of the animal outside of the shock tube, isolated from the blast wave (i.e., a torso-only exposure). Then, we developed three-dimensional (3-D) FSI models of the neck and cerebral vasculature of a rat, and used measurements from the shock-tube experiments as inputs to the models to characterize the potential blood surge to the brain caused by a torso-only exposure. Finally, we developed a 3-D finite element (FE) model of the brain, and used the brain-vasculature pressures computed from the FSI models as input to the FE model to quantify the biomechanical responses (e.g., the strain) in the tissues surrounding the brain vasculature.

## Materials and Methods

### Shock-Tube Experiments

We performed shock-tube experiments at BOPs of 70 and 130 kPa on 10-week old (330–350 g) male Sprague-Dawley rats (Charles River Laboratories, Wilmington, MA, United States). The Institutional Animal Care and Use Committee (IACUC) at the New Jersey Institute of Technology (NJIT) (Newark, NJ, United States), as well as the Animal Care and Use Review Office (ACURO) of the U.S. Army Medical Research and Development Command (USAMRDC) (Ft. Detrick, MD, United States), approved all experimental protocols related to the shock-tube experiments.

**FIGURE 1:**
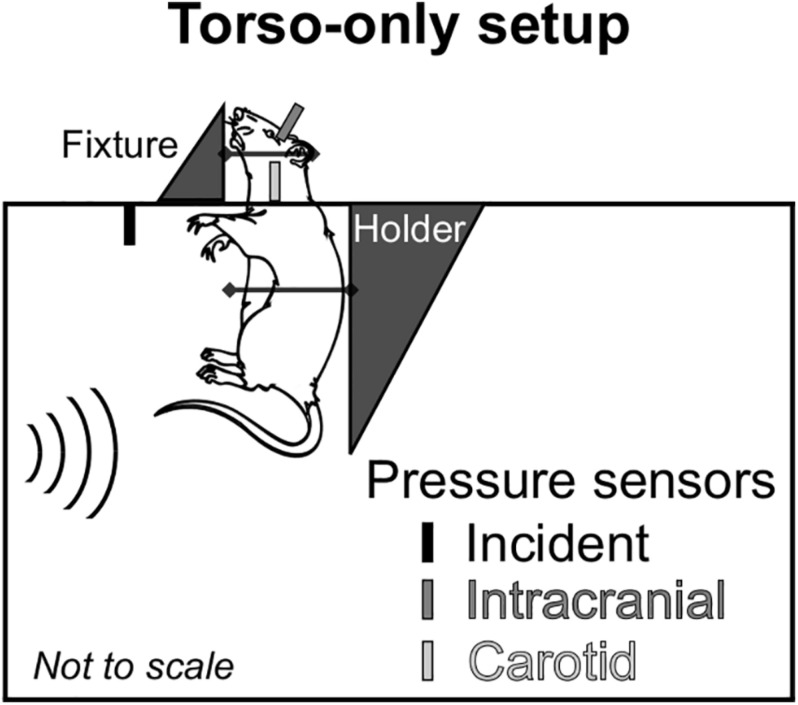
Schematic representation of the animal setup inside the shock tube designed to isolate the brain from the blast wave and assess the effect of the indirect mechanism of blast injury. We conducted all experiments with the rat in a vertical orientation, with its ventral surface facing the incident blast wave. We considered a torso-only blast-exposure configuration, wherein we isolated the head of the rat from the blast wave by keeping it outside of the shock tube and securing it to a metal fixture using Velcro straps. To minimize the motion of the animal during blast exposure, we secured the torso of the animal to a holder using a thin cotton cloth and Velcro straps. For all experiments, we measured the incident, intracranial, and carotid-artery pressures at the locations shown in the schematic.

To delineate the indirect mechanism, we exposed rats to a single torso-only blast (*n* = 8 per BOP), during which we limited the blast exposure exclusively to the torso of the rat by keeping the head outside of the shock tube (Figure 1). To enhance the blast-wave interaction with the body of the animal, we performed all experiments with the animal positioned in a vertical orientation with the ventral surface facing the incident blast wave. To conduct these experiments, we used the compressed-gas shock tube located at the NJIT Center for Injury Biomechanics, Materials, and Medicine (Kuriakose et al., 2016). Briefly, the shock tube consisted of 552- and 6,000-mm-long driver and driven sections, respectively, separated by Mylar membranes. The driven section housed a test section with a square cross-sectional area of 228 mm × 228 mm. Using a customized experimental setup, we placed the animal inside the test section of the shock tube with the head protruding through an opening in the upper wall (Figure 1). We restrained the head using Velcro straps against a vertical fixture, and mounted the torso on a holder secured by a thin cotton cloth and Velcro straps. We placed the animal at a distance of 3,080 mm from the Mylar membranes.

During the experiments, we measured the temporal profile of the static pressure produced by the blast wave at a distance of 2,692 mm from the membrane, using a pressure sensor (model 134A24; PCB Piezotronics, Depew, NY, United States) with its probe oriented parallel to the flow of the blast wave. In addition, we measured the intracranial pressure at the lateral ventricle and the intravascular pressure at the common carotid artery (CCA) using Millar pressure-catheter sensors (models SPR-407 and SPR-671, respectively; ADInstruments, Colorado Springs, CO, United States). These last two sensors were located outside of the shock tube (Figure 1). We recorded the data at a sampling frequency of 1.0 MHz.

#### Pressure Sensor Implantation

First, we anesthetized each animal using an intraperitoneal injection of 10:1 ketamine–xylazine mixture (100 mg/kg). Next, we mounted the animal on a stereotaxic frame of a Leica Angle Two computer-assisted stereotaxic system (Leica Microsystems Inc., Buffalo Grove, IL, United States). We proceeded by cutting a small section of the scalp to expose the skull. We then drilled a small hole (2 mm diameter) in the right frontal bone (−1.40 mm relative to Bregma) and slowly inserted a sterilized stainless steel guide cannula (18 gauge, 10 mm long) into the brain until the tip of the cannula reached the right lateral ventricle. We confirmed the location of the tip of the cannula using a tracking feature integrated with a built-in rat brain atlas from the stereotaxic system. Then, we anchored the cannula to the frontal bone using Loctite glue and proceeded to close the wound. Finally, we inserted a miniature Millar pressure-catheter sensor (model SPR-407, ADInstruments) into the cannula.

After implanting the intracranial pressure sensor and with the animal under anesthesia, we made a 30-mm incision in the anterior section of the neck to expose the right CCA and the vagus nerve. Next, we carefully separated the artery from the nerve and clamped the artery using two vessel clips and two loosely tied sutures. Then, we performed a V-cut on the artery and inserted a Millar pressure-catheter sensor (model SPR-671, ADInstruments). Next, we tied the sutures around the artery and the catheter, and proceeded to close the surgery wound. Finally, with the intracranial and carotid artery pressure sensors implanted, we secured the animal to the experimental holder in the shock tube. After securing the animal, we monitored the sensor measurements for 10 min to validate the readouts.

### Computational Models of the Neck and Cerebral Vasculature of a Rat

#### Geometries and Finite Element Meshes

We obtained the geometry and created a 3-D FE mesh of the neck vasculature of a rat in three steps. First, following anesthetization with isoflurane, we performed a time-of-flight magnetic resonance angiography (MRA) (7 Tesla Bruker BioSpec scanner, Bruker BioSpin Corporation, Billerica, MA, United States) scan of the neck vasculature at a uniform resolution of 127 μm per voxel. We conducted this procedure at the University of Utah Small Animal Imaging Facility (Salt Lake City, UT, United States). All imaging procedures were approved by the IACUC at the University of Utah as well as the ACURO of the USAMRDC.

Second, we imported the MRA images into 3-Matic (Materialise, Leuven, Belgium) and segmented them using a semi-automated approach to create an initial geometry of the neck vasculature. Then, we manually improved the initial geometry by removing discontinuities and smoothing sharp angles. This neck geometry originated at the CCA and included the internal carotid, external carotid, and vertebral arteries, as well as the initial segments of the arteries forming the circle of Willis (CoW) at the base of the brain. Lastly, we imported the improved geometry into Hypermesh 2017.1 (Altair Engineering, Troy, MI, United States) and meshed the geometry using 171,892 linear triangular (three-noded) shell elements of type S3. We meshed the neck vasculature with shell elements having an average minimum edge length of 0.088 mm.

We obtained the geometry and developed a 3-D FE mesh of the major cerebral vasculature of a rat based on micro-computed tomography (μCT) images (Unnikrishnan et al., 2019). Briefly, we perfused the brain with a high-contrast setting agent and performed a μCT scan of the rat brain at a uniform resolution of 35 μm per voxel. The detailed protocols for the administration of the high-contrast agent and the μCT scan are described in our previous work (Unnikrishnan et al., 2019). From the μCT images of the rat brain with the contrast agent, we segmented and improved the initial geometry of the cerebral vasculature of a rat using the same approach as that described above for the neck vasculature. Then, we identified the major vessels and created a unified vascular network. This cerebrovascular network originated at the base of the brain and included, among others, the basilar artery (BA) and the arteries forming the CoW. In addition, it included the major veins, such as the superior sagittal sinus and transverse sinus. The cerebrovascular network consisted of vessels with a diameter larger than 0.13 mm. Using Hypermesh, we meshed the geometry of the cerebral vasculature using 217,827 linear triangular (three-noded) shell elements of type S3, with an average minimum edge length of 0.063 mm.

We selected the thickness of the vessels based on literature data, which showed that the thickness-to-diameter ratio in rat vessels ranges from 0.1 to 0.3 (Harper and Bohlen, 1984; Vovenko, 1999). Considering an average diameter of 0.5 mm, we calculated the thickness to vary between 0.05 and 0.15 mm. Hence, we set the cerebral and neck vasculature thicknesses to 0.05 and 0.10 mm, respectively. In addition, because we did not have the spatial variation in wall thickness for the vessels, we set the vessels to have a uniform thickness.

#### Computational Fluid Dynamics Meshes

Using Hypermesh and the above-described FE meshes as templates, we developed 3-D computational fluid dynamics (CFD) meshes of the blood-filled lumina of the neck and cerebral vasculature of a rat. To this end, we used 663,979 and 532,243 tetrahedral (four-noded) elements of type FC3D4 to generate the CFD meshes of the neck (minimum edge length = 0.088 mm) and the cerebral (minimum edge length = 0.068 mm) vasculature, respectively.

#### Material Properties

We considered the neck and cerebral vasculature as incompressible, isotropic, hyperelastic materials. We used a one-term Ogden model to capture the deviatoric response of the vasculature wall (Unnikrishnan et al., 2019). We obtained these properties (Table 1) from high-strain-rate axial testing of the middle cerebral arteries of male Sprague-Dawley rats (Bell et al., 2018). Because these experiments showed no difference between the response of arteries tested at quasi-static conditions and those tested at high strain rates relevant to blast exposure (<500 s^–1^), we did not account for the viscoelastic behavior of the vessels in our material model.

**TABLE 1:** Material properties of the different components of the finite element models of the neck vasculature, cerebral vasculature, and brain of a rat (Unnikrishnan et al., 2019).

Components	Density	Hyperelastic constants			Viscous constants	
	(kg/m^3^)	Bulk modulus (GPa)	Shear modulus (kPa)	α	Modulus ratio	Relaxation time (ms)
Vasculature	1040	2.1	685.0	4.3		
Cerebrum	1040	2.1	11.9	6.5	0.897	1.01
Cerebellum	1040	2.1	8.3	8.2	0.726	2.49
Brainstem	1040	2.1	12.3	4.7	0.888	0.93

Considering that we did not include microvessels (i.e., arterioles, venules, and capillaries) in our model, we represented the blood flowing through the lumina of the neck and cerebral vasculature as a Newtonian fluid. Previous studies demonstrated the suitability of the Newtonian approximation for modeling blood flow in large vessels (Ambrosi et al., 2012; Sriram et al., 2014). Moreover, we set the set a fluid density of 1030 kg/m^3^ and a viscosity of 0.0035 kg/m⋅s (Wang et al., 2001; Ambrosi et al., 2012).

#### Fluid-Structure Interaction Model of the Neck Vasculature of a Rat

**FIGURE 2:**
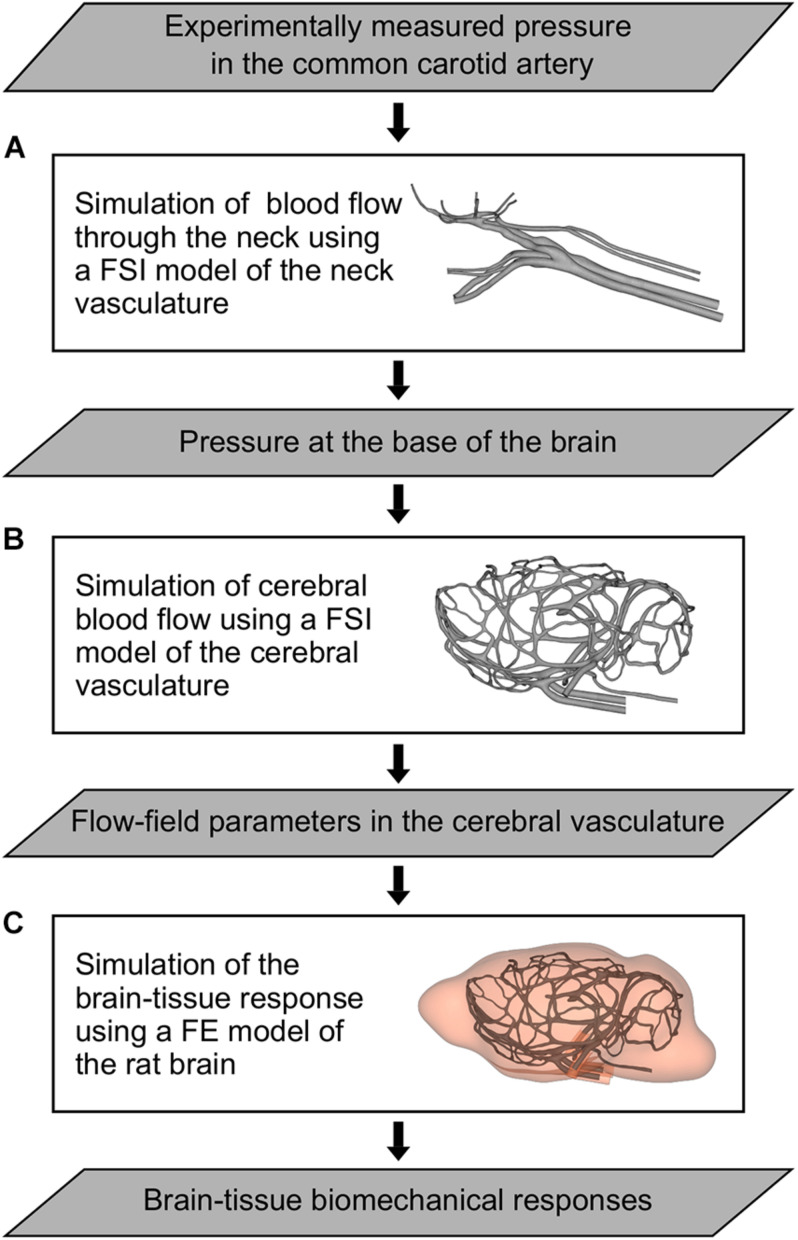
Flowchart of the computational models and simulations performed to characterize the indirect mechanism of blast injury in a rat. **(A)** Fluid-structure interaction (FSI) model of the neck vasculature developed to simulate the blood flow through the neck and to characterize the pressure propagation in the neck vessels (i.e., from the common carotid artery to the internal carotid artery) for a torso-only blast condition. **(B)** FSI model of the cerebral vasculature developed to simulate the cerebral blood flow and to characterize the flow-field parameters (e.g., the mass flow rate, pressure, velocity, and wall shear stress) in the cerebral vessels for torso-only blast and blast-free (i.e., normotensive) conditions. **(C)** Finite element (FE) model of the rat brain developed to predict brain-tissue biomechanical responses (e.g., the strain) resulting from a torso-only exposure.

We developed a 3-D FSI model of the neck vasculature of a rat (Figure 2A) by coupling the corresponding FE and CFD models using the co-simulation feature in Abaqus v6.14 (Dassault Systèmes, Vélizy-Villacoublay, France).

**FIGURE 3:**
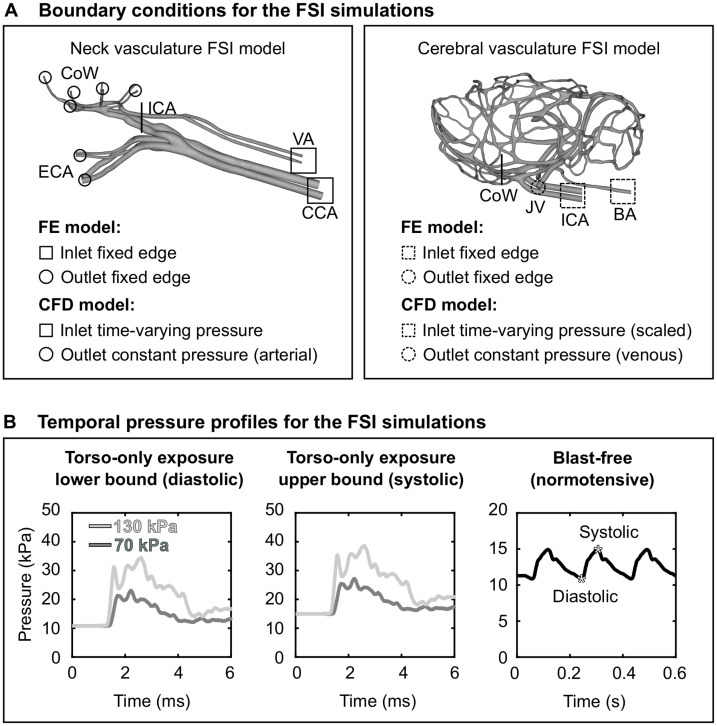
Boundary conditions for the fluid-structure interaction (FSI) simulations of the neck and cerebral blood flow in a rat. **(A)** Boundary conditions defined in the finite element (FE) and computational fluid dynamics (CFD) models of the neck vasculature (left panel) and cerebral vasculature (right panel) and used in their corresponding FSI simulations. **(B)** Temporal pressure profiles used as inlet boundary conditions for torso-only blast (left and center panels) and blast-free [right panel (Cosson et al., 2007)] conditions. BA, basilar artery; CCA, common carotid artery; CoW, circle of Willis; ECA, external carotid artery; ICA, internal carotid artery; JV, jugular vein; VA, vertebral artery.

As a boundary condition for the FE model of the neck vasculature, we set the displacement to zero at the free edges from both the inlet vessels [i.e., the CCA and the vertebral artery (VA)] and the outlet vessels [i.e., the external carotid artery (ECA) and the CoW arteries; Figure 3A, left panel]. Similar to other researchers (Liu et al., 2007), we applied a uniform pressure of 4,000 Pa on the external wall of the neck vessels to account for the surrounding tissues. For the CFD model of the neck vasculature, we specified a time-dependent pressure boundary condition at the inlet surfaces. Moreover, we defined a constant pressure (either the systolic or the diastolic pressure of a cardiac cycle of a rat) at the outlet surfaces of the CFD model of the neck vasculature.

#### Fluid-Structure Interaction Model of the Cerebral Vasculature of a Rat

As we did for the FSI model of the neck vasculature, we developed the FSI model of the cerebral vasculature of a rat (Figure 2B) by coupling the corresponding FE and CFD models using the co-simulation feature in Abaqus. As a boundary condition for the FE model of the cerebral vasculature, we set the displacement for both the inlet vessels [i.e., the internal carotid artery (ICA) and the BA] and the outlet vessels [i.e., the jugular veins (JV); Figure 3A, right panel)] to zero at the free edges. Similar to previous work (Liu et al., 2007), we applied a uniform pressure on the external wall of the cerebral vessels to account for the surrounding tissues. We set this pressure to 1,333 Pa, which is equivalent to the intracranial pressure of a normotensive Sprague-Dawley rat (Bragin et al., 2013). For the CFD model of the cerebral vasculature, we specified a scaled-down time-dependent boundary condition at the inlet surfaces, which we determined using the carotid-artery pressure measurement and the neck vasculature model. First, we considered the pressure measurement as the input to the neck model to compute the pressure drop between the CCA and the ICA. Then, we used this pressure drop as a constant to linearly scale down the measured pressure profile at the CCA, and used this scaled-down pressure profile as the input to the cerebral vasculature model. In addition, we set the pressure at the outlet surfaces of the CFD model of the cerebral vasculature to 1,000 Pa, which corresponds to the venous pressure of a normotensive Sprague-Dawley rat (Schierling et al., 2009).

### Neck Blood Flow Simulations

Using the FSI model of the neck vasculature, we performed simulations to characterize the blood flow through the neck of a rat for torso-only blast conditions at incident BOPs of 70 and 130 kPa. To perform these simulations, we used the temporal profile of the carotid-artery pressure measurements obtained for each incident BOP (Figure 3B, left and middle panels) as the inlet boundary condition. For each BOP, we simulated two limiting conditions: a lower-bound condition, where the baseline pressure was the diastolic pressure (10.8 kPa) of a normotensive cardiac cycle of a rat, and an upper-bound condition, where the baseline pressure was the corresponding systolic pressure (14.9 kPa). For all four conditions (i.e., the lower- and upper-bound conditions for a torso-only exposure of 70 kPa and the corresponding conditions for an exposure of 130 kPa), we characterized the pressure propagation along the neck vessels.

### Cerebral Blood Flow Simulations

Using the FSI model of the cerebral vasculature, we performed simulations to characterize the cerebral blood flow for the same BOPs and the same limiting conditions described above for the FSI model of the neck vasculature. In addition, we simulated a blast-free rat normotensive physiological condition considering the cardiac cycle detailed by Cosson et al. (2007), with a duration of 0.185 s and a mean arterial pressure of 12.2 kPa (Figure 3B, right panel). For all five conditions (i.e., the lower- and upper-bound conditions for a torso-only exposure of 70 kPa, the corresponding conditions for an exposure of 130 kPa, and the blast-free condition), we computed and compared the mass flow rate, pressure, velocity, and wall shear stress in the cerebral vessels. We also quantified the influence of vasculature thickness on the FSI predictions by assigning a thickness of 0.035 or 0.065 mm to all vasculature elements, in addition to the selected nominal value of 0.05 mm.

### Finite Element Model of the Rat Brain

#### Finite Element Meshes

We developed a 3-D FE model of the rat brain (Figure 2C) by coupling the FE mesh of the cerebral vasculature of a rat with a modified version of the FE mesh of the rat brain previously described by Unnikrishnan et al. (2019). Briefly, we modified the rat brain mesh reported by Unnikrishnan et al. (2019) to include the cavities of the major cerebral vessels, which resulted in approximately 2.6 million quadratic tetrahedral (10-noded) elements of type C3D10M. In doing so, we made sure that the modified mesh of the rat brain matched element-by-element the mesh of the cerebral vasculature. Then, we coupled the modified FE mesh of the rat brain with the FE mesh of the cerebral vasculature using a tie constraint, wherein we only constrained the translational degrees of freedom of the brain and vasculature elements in all directions.

#### Material Properties

We represented the brain as an incompressible, hyper-viscoelastic material, using a one-term Ogden model with a one-term Prony series (Unnikrishnan et al., 2019). We obtained the material properties of the brain (Table 1) from high-strain-rate shear tests performed on samples from the cerebrum, cerebellum, and brainstem regions of male Sprague-Dawley rats (Haslach et al., 2017).

#### Brain-Tissue Response Simulations

For each cerebral blood flow simulation conducted using the FSI model of the cerebral vasculature, we extracted the pressure field at the internal surface of the cerebral vasculature and used these pressures as the boundary condition in the FE model of the rat brain. We did not apply any pressure on the outer walls of the cerebral vasculature. For all conditions (i.e., the lower- and upper-bound conditions for a torso-only exposure of 70 kPa, the corresponding conditions for an exposure of 130 kPa, and the blast-free condition), we computed and compared the biomechanical responses (e.g., the strain) of the brain tissue.

## Results

### Blast-Wave Interaction With the Torso

**FIGURE 4:**
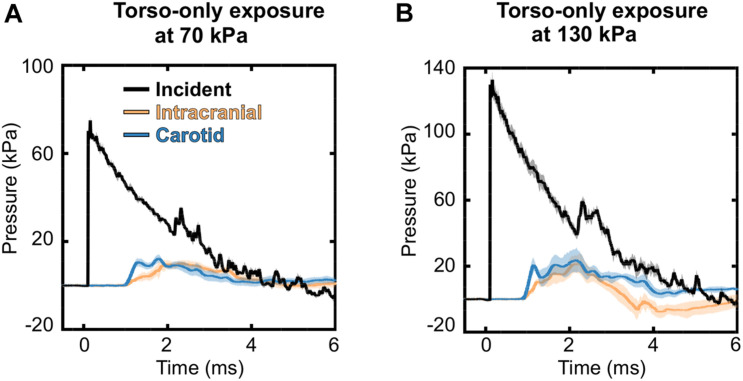
Temporal profiles of the incident, intracranial, and carotid-artery pressures for the torso-only blast-exposure experiments at incident blast overpressures of **(A)** 70 kPa and **(B)** 130 kPa. The solid lines and shaded areas represent the mean (*n* = 8) and one standard deviation, respectively. (Because the standard deviations are small, they may not be visible).

To characterize the interaction of a blast wave with the torso of a rat, we conducted torso-only blast-exposure experiments in a shock tube. For the targeted incident BOP of 70 kPa, the measured peak overpressures were 75.10 ± 1.82 kPa [mean ± one standard deviation (SD)] for the static incident pressure inside the tube, 12.21 ± 1.26 kPa for the intracranial pressure, and 13.14 ± 1.73 kPa for the carotid-artery pressure (Figure 4A). The corresponding measurements for the targeted 130 kPa BOP were 133.16 ± 5.12 kPa, 24.31 ± 6.61 kPa, and 28.94 ± 5.45 kPa, respectively (Figure 4B). For both BOPs, the temporal profiles of the incident pressure showed a nearly instantaneous rise to the peak overpressure, followed by a rapid non-linear decay, and a subsequent return to baseline conditions. In contrast, the intracranial and carotid-artery pressures rose after a ∼1.2-ms delay to considerably lower peak overpressures and showed attenuated non-linear decays (Figures 4A,B). Table 2 shows other relevant blast-wave parameters, such as impulse and duration.

**TABLE 2:** Summary of relevant blast-wave parameters of the torso-only exposure experiments.

Pressure measurement	Parameter		
	Peak overpressure (kPa)	Duration (ms)	Impulse (kPa⋅ms)
**Blast 70 kPa**			
Incident	75.10 ± 1.82	4.22 ± 0.10	119.77 ± 2.21
Carotid artery	13.14 ± 1.73	5.07 ± 2.54	23.76 ± 9.40
Intracranial	12.21 ± 1.26	3.40 ± 0.77	19.04 ± 1.23
**Blast 130 kPa**			
Incident	133.16 ± 5.12	5.20 ± 0.04	236.16 ± 12.58
Carotid artery	28.94 ± 5.45	5.14 ± 1.60	55.97 ± 12.55
Intracranial	24.31 ± 6.61	2.39 ± 0.19	26.40 ± 4.67

#### Effect of Shock-Tube Walls on the Pressure Field

To determine whether reflected pressure waves or uneven pressure fields caused by the shock-tube walls could have affected the load to the torso of the rat, we compared thoracic-pressure measurements (at the carotid artery) taken inside of the shock tube between torso-only and whole-body exposures. For the torso-only exposures, we used the experimental setup and shock tube described above in Materials and Methods. In contrast, for the whole-body exposures, we used a larger shock tube with a square cross-sectional area of 711 mm × 711 mm (Alay et al., 2018), wherein we placed the entire animal inside the test section of the shock tube away from the side walls. When comparing the thoracic pressures between the two configurations, the measurements had similar peak overpressures and temporal profiles (Supplementary Figure 1). This similarity suggests that the shock-tube walls did not influence the load to the torso of the rat in the torso-only exposure.

### Characterization of the Neck and Cerebral Blood Flows

Before running the FSI simulations for the neck vasculature, we performed mesh-convergence tests of the coupled FE and CFD vasculature models to determine the adequate number of mesh elements. To this end, while considering a lower-bound torso-only exposure of 70 kPa, we systematically increased the number of mesh elements in the two models and evaluated the changes in pressure and wall shear predictions. The peak pressure predicted at the ICA (ICA in Figure 3A, left panel) by the current model (N3 in Table 3) increased marginally (<1%) when we increased the number of mesh elements, indicating the convergence of the results. Moreover, the difference in wall shear stress between the current model and a model with 41% additional mesh elements was approximately 5%. A similar analysis for the cerebral vasculature model indicated that the peak pressure at the CoW (CoW in Figure 3A, right panel) and the wall shear stress predicted by the current model (C3 in Table 3) were only 1 and 4% different, respectively, when we increased the number of mesh elements, demonstrating the convergence of the results.

**TABLE 3:** Summary of the mesh convergence tests performed on the FSI models of the neck and cerebral vasculature of a rat.

Model	Number of elements		Peak pressure (kPa)	Average of wall shear stress (Pa)
	FE	CFD		
**Neck vasculature**				
N1	91,409	339,612	17.11	44.78
N2	118,812	481,429	17.40	48.23
N3*	171,892	663,979	17.64	52.35
N4	206,198	975,474	17.67	54.90
**Cerebral vasculature**				
C1	97,346	178,142	14.11	20.12
C2	155,494	349,650	14.15	24.33
C3*	217,827	532,243	14.29	27.01
C4	242,937	663,085	14.31	28.18

*Selected. CFD, computational fluid dynamics; FE, finite element; FSI, fluid-structure interaction.

**FIGURE 5:**
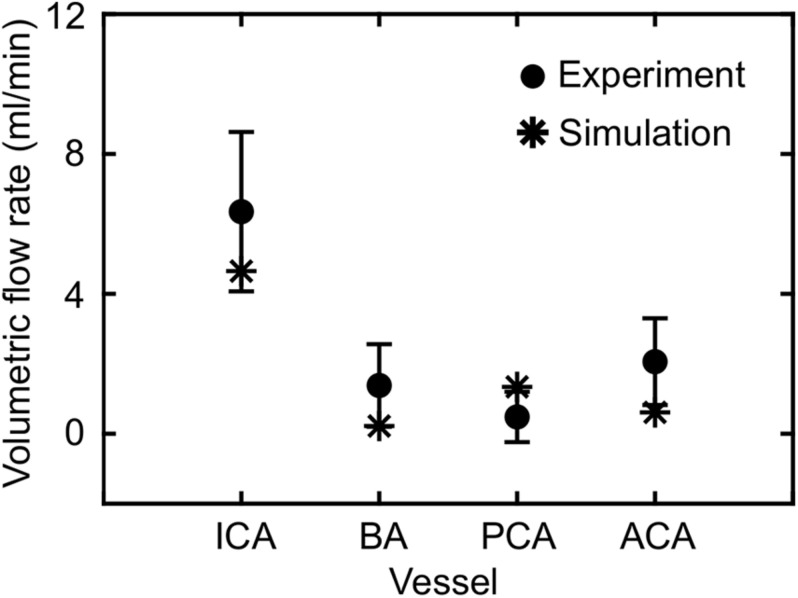
Comparison of the volumetric flow rate in different vessels of the cerebral vasculature of a rat predicted by the fluid-structure interaction model for the blast-free, normotensive condition with experimental data (Schierling et al., 2009). The solid circles and error bars represent the mean (*n* = 10) and two standard errors of the mean, respectively, averaged over one cardiac cycle. The asterisks indicate model predictions. ACA, anterior cerebral artery; BA, basilar artery; ICA, internal carotid artery; PCA, posterior cerebral artery.

Based on the FSI simulations of the neck vasculature, we characterized the blood flow and pressure propagation through the neck vessels of a rat resulting from the torso-only exposure. Compared to the peak overpressure predicted at the CCA (CCA in Figure 3A, left panel), our simulations predicted a 46% reduction of the peak overpressure in the ICA at the base of the brain (ICA in Figure 3A, left panel), when we averaged over the four conditions (Table 4). Similarly, we performed FSI simulations of the cerebral vasculature and characterized its blood flow by computing flow-field parameters, such as the mass flow rate, pressure, velocity, and wall shear stress. To validate the model predictions, we compared the experimental measurements of the volumetric flow rates in different cerebral vessels (Schierling et al., 2009) with the model predictions for a blast-free, normotensive condition, and found them to be in very good agreement (Figure 5). Specifically, averaged over one cardiac cycle, the model-predicted volumetric flow rates for the internal carotid (4.65 ml/min), basilar (0.22 ml/min), posterior cerebral (1.34 ml/min), and anterior cerebral (0.61 ml/min) arteries each fell within two standard errors of the mean (SEM) experimental values [internal carotid: 6.35 ± 2.28 ml/min; basilar: 1.38 ml/min ± 1.18 ml/min; posterior cerebral: 0.48 ± 0.72 ml/min; and anterior cerebral: 2.03 ± 1.24 ml/min (mean ± 2 SEM)], suggesting that the model predictions are indistinguishable from the experimental data.

**TABLE 4:** Pressure propagation through the neck vessels of a rat exposed to a torso-only blast.

Condition	Peak overpressure (kPa)	
	CCA	ICA
**Blast 70 kPa**		
Lower bound	12.25	6.84
Upper bound	12.24	5.04
**Blast 130 kPa**		
Lower bound	23.65	15.94
Upper bound	23.65	12.45

*CCA, common carotid artery; ICA, internal carotid artery.*

For the blast-free and torso-only blast conditions, we computed and compared the mass flow rates at the base of the brain to assess whether a torso-only exposure could generate a noticeable blood surge to the brain. For the blast-free condition, the FSI simulations predicted the peak mass flow rate entering the rat brain to be 20.45 ml/min. In contrast, for the torso-only exposure of 70 kPa, the predicted peak mass flow rates were 51.68 and 53.77 ml/min for the lower- and upper-bound conditions, respectively. Similarly, the predicted peak mass flow rates for the corresponding conditions at 130 kPa were 68.45 and 72.62 ml/min, respectively.

To further characterize the blood surge to the brain, we also determined the velocity field in the cerebral vasculature. Notably, even for the lower-bound condition of the 70 kPa BOP exposure, relative to the blast-free condition, we observed increases in the peak velocities in different vessels at the base of the brain that ranged from 59 to 184% (Table 5). Consistent with these changes, the predicted peak velocities in the same vessels for the 130 kPa exposure were substantially higher.

**TABLE 5:** Comparison of the predicted peak velocities at different cerebral arteries of a rat brain, for blast-free and torso-only blast conditions.

Condition	Peak velocity (cm/s)			
	PCA	MCA	ACA	AZA
Blast-free (normotensive)	43.22	27.66	17.65	12.04
**Blast 70 kPa**				
Lower bound	68.69	65.84	50.12	33.43
Upper bound	75.69	72.70	52.84	36.23
**Blast 130 kPa**				
Lower bound	101.55	103.60	74.29	56.50
Upper bound	108.28	108.65	75.11	56.85

*ACA, anterior cerebral artery; AZA, azygos artery; MCA, middle cerebral artery; PCA, posterior cerebral artery.*

**FIGURE 6:**
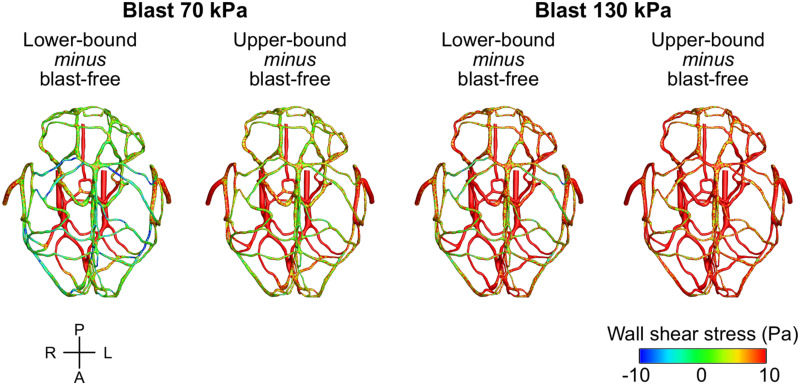
Differences in the peak wall shear stress in the cerebral vasculature of a rat brain between the blast-free, normotensive condition and the torso-only blast conditions. For each pair of conditions, we computed their differences by first determining the peak wall shear stress for each condition at each shell element of the cerebral vasculature (over one cardiac cycle for the blast-free condition and over the blast-exposure simulation time for the torso-only conditions), and then subtracted them. A, anterior; L, left; P, posterior; R, right.

To characterize the effect of the blood surge on the cerebral vasculature, we determined and compared the wall shear stresses on the vessels at three different brain regions: the cerebrum, cerebellum, and brainstem. For all five conditions (i.e., one blast-free and four torso-only exposures), the average values of the peak wall shear stress differed considerably across the three brain regions, with the highest values observed in the cerebrum (Table 6). Compared to the blast-free condition, the average peak wall shear stress increased by 20 to 90% in the cerebrum, by 2 to 111% in the cerebellum, and by 153 to 289% in the brainstem for the torso-only exposures. In addition, to further compare the peak wall shear stress on the cerebral vasculature between the blast-free and torso-only blast conditions, we computed their difference maps (Figure 6). Relative to the blast-free condition, the maps for each of the four blast conditions revealed major differences at the base of the brain. In contrast, for the 70 kPa exposure, the vessels located at the peripheral regions of the brain showed smaller differences (Figure 6, left panels). In addition, compared to the blast-free condition, the wall shear stresses for the 130 kPa exposure were considerably higher throughout the brain (Figure 6, right panels).

**TABLE 6:** Comparison of the predicted peak wall shear stress for different regions of the rat brain, for blast-free and torso-only blast conditions.

Condition	Average of the peak wall shear stress (Pa)		
	Cerebrum	Cerebellum	Brainstem
Blast-free (normotensive)	20.83	6.32	6.34
**Blast 70 kPa**			
Lower bound	25.02	6.46	16.02
Upper bound	30.67	7.91	17.72
**Blast 130 kPa**			
Lower bound	35.26	11.40	23.33
Upper bound	39.59	13.33	24.69

To evaluate the effect of vasculature thickness on the FSI model predictions, we parametrically varied the cerebral vasculature thickness between 0.035 and 0.065 mm and performed simulations for lower-bound torso-only exposures of 70 and 130 kPa. We observed that, as the vasculature thickness decreased, the inlet peak mass flow rate and the pressure at the ICA decreased by as much as 6% (Table 7). In contrast, the average values of the peak wall shear stress did not change consistently with changes in the wall thickness.

**TABLE 7:** Comparison of flow-field parameters for the cerebral vasculature model for different vasculature thicknesses.

Condition	Vasculature thickness	Flow-field parameter				
		Peak mass flow rate (ml/min)	Peak pressure at CoW (kPa)	Average peak of WSS in cerebrum (Pa)	Average peak of WSS in cerebellum (Pa)	Average peak of WSS in brainstem (Pa)
**Blast 70 kPa**						
	0.035	49.16	13.67	24.46	5.74	16.28
	0.050	51.68	14.29	25.02	6.46	16.02
	0.065	51.80	14.43	25.04	5.59	15.82
**Blast 130 kPa**						
	0.035	64.64	20.78	34.46	10.69	22.74
	0.050	68.45	21.51	35.26	11.40	23.33
	0.065	68.78	22.04	35.47	11.30	23.32

*CoW, circle of Willis; WSS, wall shear stress.*

### Characterization of the Brain-Tissue Response

**FIGURE 7:**
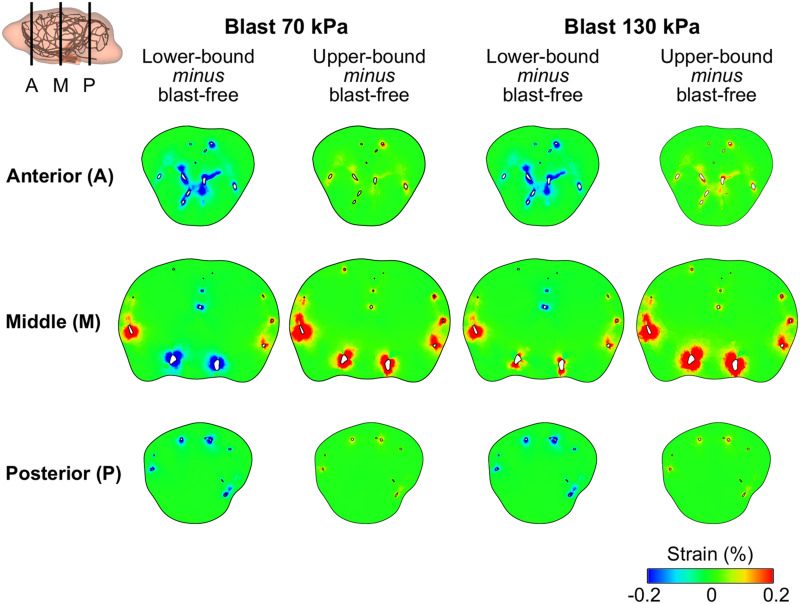
Differences in the peak maximum principal strain in the brain tissue of a rat between the blast-free, normotensive condition and the torso-only blast conditions. The differences maps are for the coronal plane at the anterior, middle, and posterior regions of the rat brain. For each pair of conditions, we computed their differences by first determining the maximum strain for each condition at each tetrahedral element of the rat brain (over one cardiac cycle for the blast-free condition and over the blast-exposure simulation time for the torso-only conditions), and the subtracted them.

Using the FE model of the rat brain, we characterized the biomechanical responses of the tissues in the brain resulting from a torso-only exposure. To conduct these simulations, we determined the pressure fields at the internal surface of the cerebral vasculature from the FSI simulations and used them as the loading pressure boundary condition for the FE brain model. Because the normal forces due to the blood pressure are transferred across the wall thickness (Papaioannou and Stefanadis, 2005), we selected the fluid pressure as the boundary condition for the FE simulations. For each of the five conditions (i.e., one blast-free and four torso-only exposures), we computed the peak maximum principal strain in the cerebrum, cerebellum, and brainstem by averaging the largest values within a region over one cardiac cycle for the blast-free condition and over the blast-exposure simulation time for the torso-only conditions. When compared to the blast-free condition, the two upper-bound conditions yielded values that were as much as 16% higher (Table 8). In contrast, the two lower-bound conditions revealed average strains that were as much as 20% lower than the normotensive condition. Importantly, the absolute magnitude of the strain differences for each of the comparisons was negligible (less than 1%). In addition, we computed difference maps for the coronal plane at the anterior, middle, and posterior regions of the brain (Figure 7). As expected, the differences were larger in the tissues surrounding the cerebral vessels, and were primarily positive in magnitude for the upper-bound conditions. In contrast to the middle region, differences at the anterior and posterior regions of the brain were predominantly negative for the lower-bound conditions.

**TABLE 8:** Comparison of the predicted peak maximum principal strain for different regions of the rat brain, for blast-free and torso-only blast conditions.

Condition	Average of the peak maximum principal strain (%)		
	Cerebrum	Cerebellum	Brainstem
Blast-free (normotensive)	0.57	0.60	0.36
**Blast 70 kPa**			
Lower bound	0.50	0.48	0.29
Upper bound	0.65	0.63	0.38
**Blast 130 kPa**			
Lower bound	0.56	0.50	0.30
Upper bound	0.66	0.65	0.39

## Discussion

The potential role of the indirect mechanism in causing blast-induced TBI remains inconclusive. To address this knowledge gap, first, we conducted shock-tube experiments on male Sprague-Dawley rats. Using a torso-only configuration to characterize the interaction of the blast wave with the torso, we measured the intracranial and carotid-artery pressures, while keeping the head of the animal outside of the shock tube (Figure 1). Then, we developed and validated 3-D FSI models of the neck and cerebral vasculature. Using the experimental data as input to the models, we determined whether a torso-only exposure could generate a blood surge to the brain and evaluated the extent to which the surge would increase the wall shear stress in the cerebral vasculature. Finally, we developed a 3-D FE model of a rat brain. Using the pressure fields at the internal surface of the cerebral vasculature computed by the FSI models to drive the FE simulations, we characterized the brain-tissue responses resulting from a torso-only exposure (Figure 2).

In our experimental configuration, we isolated the rat’s head by placing it outside of the shock tube, while maximizing exposure to the torso by placing the ventral surface of the animal facing the blast wave. Initially, we attempted to isolate the head of the animal using a head-shielded configuration using either steel or a thermoplastic polymer (ABS) as the shielding material. In this configuration, we placed the animal in a prone orientation facing the incident blast wave and simultaneously measured the incident pressure and the pressure on the forehead of the animal under the shielding (Supplementary Figure 2). Interestingly, we found that the pressure profile measured on the forehead largely overlapped with the incident-pressure profile with a ∼1-ms delay, and that the shielding attenuated the peak pressure by only 33% for steel and 22% for ABS (Supplementary Figure 3). In stark contrast, in our torso-only configuration with the head placed outside of the shock tube, when we compared the pressure on the surface of the animal (at the collar) with the incident pressure, we observed a considerable attenuation of the temporal profile and an 84% reduction in the peak pressure (Supplementary Figure 3). These results strongly suggest that to effectively isolate the brain from a blast wave in a shock tube, we must place the head of the animal outside of the tube.

Relative to its baseline value, a torso-only exposure of 70 and 130 kPa caused a very modest increase in the intracranial pressure (12.21 to 24.31 kPa; Figure 4 and Table 2) and resulted in a substantially attenuated temporal pressure profile. This is markedly different from the results for a whole-body rat exposure in the prone orientation, where the temporal profile of the intracranial pressure tracked and largely overlapped with that of the incident pressure and the peak overpressures were comparable in magnitude (Leonardi et al., 2011; Sundaramurthy et al., 2012).

The pressure measurements at the carotid artery (outside of the shock tube) indicate that the pressure wave propagates from the torso through the neck, although with modest peak overpressures (13.14 to 28.94 kPa; Table 2 and Figure 4). Using these pressure profiles as inputs, we used the 3-D FSI models of the neck and cerebral vasculature to determine whether a torso-only exposure could generate a blood surge to the brain and to evaluate the extent to which the surge would increase the wall shear stress in the rat’s cerebral vasculature. When we computed the mass flow rate at the base of the brain for the 70 and 130 kPa BOP exposures and compared them with the blast-free, normotensive condition, we observed a 2.6- to 3.5-fold increase in their maximum values, respectively. This increase led to the propagation of the blood surge from the base of the brain to the cerebrovascular network, where we predicted a considerable increase in the velocity fields at different cerebral vessels (Table 5). For instance, at the middle cerebral artery, a vessel that supplies blood to the cortex and the anterior regions of the brain, relative to the blast-free condition, a torso-only exposure of 70 kPa caused as much as a 2.6-fold increase in the peak velocity and a 3.9-fold increase for the 130 kPa exposure. Together, our analyses show that a torso-only exposure causes a sudden and abundant stream of blood to rapidly propagate from the torso through the neck to the cerebral vasculature.

This blood surge in the cerebral vasculature caused a considerable increase in the wall shear stresses. Relative to the blast-free, normotensive condition, our models predicted higher average values of peak wall shear stresses throughout the brain, for all blast conditions (Table 6). As expected, the cerebrum and brainstem had higher values mainly because these regions accommodate the vessels through which the blood surge enters the brain (i.e., the arteries of the CoW and the BA, respectively) and generate high gradients in velocity. In addition, we observed attenuated differences between the 70 kPa exposure and blast-free conditions in the peak wall shear stress at the peripheral and posterior vessels of the cerebral vasculature (Figure 6), which are primarily due to the dissipation of momentum of the blood surge as it travels through the complex cerebrovascular network. Previously described as an inherent functional property of the cerebral vasculature (Vrselja et al., 2014; Fievisohn et al., 2018), this dissipation is caused by the bifurcations in the cerebral vasculature that increase the resistance to flow and ensure the decrease in blood velocity.

Using the computed pressure profiles at the internal surface of the cerebral vasculature as inputs, we used the FE model of the brain tissue to predict the strain response. Our results indicate that a torso-only exposure has an insignificant effect on the brain-tissue strain, yielding differences between the blast and blast-free conditions in the peak strain of less than 0.2% (Table 8 and Figure 7). The magnitude of these differences also depended on whether the exposure occurred during the diastolic phase or the systolic phase of the cardiac cycle. For instance, we observed higher peak strains for the upper-bound conditions of both 70 and 130 kPa BOP exposures (Table 8). In contrast, the peak strains were slightly lower for the lower-bound conditions. Interestingly, these tissue strains were much lower than the strain thresholds for tissue-level damage suggested in the literature (Bain and Meaney, 2000; Geddes et al., 2003; Zhang et al., 2004; Cater et al., 2006). Moreover, the predicted strain for a torso-only exposure is considerably lower than that for a head-only exposure. For example, for a head-only exposure of 200 kPa, Unnikrishnan et al. (2019) reported average peak strains of 5.5% at the cerebrum, whereas in our torso-only exposure of 130 kPa we predicted average peak strains of up to 0.65% (Table 8). Such a contrasting difference provides a means to quantify the marginal increase in brain-strain response resulting from a torso-only blast exposure.

Taken together, our study of the biomechanical responses of the brain of a rat resulting from a torso-only blast exposure highlights the potential structural effects, which in turn may lead to functional deficits, of the indirect mechanism on the brain vasculature. Extensive evidence from both *in vivo* and *in vitro* studies supports the notion that increases in hemodynamic stresses, such as in our predictions of elevated wall shear stresses, can alter the endothelial cells at the walls of the blood vessels (Lehoux et al., 2006; Szymanski et al., 2008; Aoki et al., 2011; Lu and Kassab, 2011; Chalouhi et al., 2012). Based on different rodent models, previous experimental studies have also reported varying degrees of acute (24 h) and chronic (6 weeks following blast exposure) vascular pathologies associated with whole-body exposure, including impairment of the vasodilation mechanisms (Rodriguez et al., 2018), permeability of the blood-brain barrier (Kuriakose et al., 2018, 2019; Heyburn et al., 2019), and structural alterations in the smooth muscle layers of the cerebral arteries (Gama Sosa et al., 2013, 2014, 2019). However, because these observations were based on a whole-body-exposure configuration, it is unknown whether these vascular pathologies are caused by the indirect mechanism, the direct mechanism, or both. Our findings underscore the need for future experimental studies to address this uncertainty and clearly delineate the role of the indirect mechanism in vascular pathology.

Our study has limitations. First, we assumed that the increase in the measured pressure at the carotid artery corresponded to an increase in the blood pressure within the vessel due to the torso-only blast exposure. However, it is possible that, to some extent, these measurements could represent blast-induced pressures that propagated through the neck tissues and vasculature. Nevertheless, multiple animal studies on cardiopulmonary resuscitation support the notion that blood pressure in the thoracic arteries increases in response to increases in intrathoracic pressure (Rudikoff et al., 1980; Weisfeldt et al., 1981; Maier et al., 1984), suggesting that our assumption linking carotid-artery pressure increases to blast-induced intrathoracic pressure increases to be valid. Second, because we did not capture the cerebral vessels with a diameter of less than 35 μm (owing to limitations of the μCT imaging), we did not include arterioles, capillary vessels, and venules in our model of the cerebral vasculature. However, as demonstrated in our validation analysis for the normotensive condition, the exclusion of these small vessels might not affect the predictions of cerebral blood flow for the major arteries and veins. Third, due to the unavailability of material properties for the cerebral veins of Sprague-Dawley rats, for the FSI model, we implemented the same material properties as those for the cerebral arteries. Considering that the majority of the flow pulse is dissipated in the arteries and not in the veins, we expect the overall findings discussed herein to remain valid despite this approximation. Finally, due to the lack of experimental data of mass flow rate, wall shear stress, and strain in the brain of a rat in response to blast exposure, we could validate our model predictions only for mass flow rates for a blast-free, normotensive condition.

## Conclusion

We performed blast-tube experiments and developed 3-D computational models of the neck vasculature, cerebral vasculature, and brain tissue to delineate the interaction of a blast wave with the torso of a rat and to quantitatively characterize the biomechanical effects at the brain vasculature and tissue levels of this potential, indirect mechanism of brain injury. Our experimental results showed that a torso-only blast exposure, in the absence of a head exposure, causes an increase in the carotid-artery pressure, supporting the notion that the interaction of the blast wave with the torso causes a sudden and abundant stream of blood to rapidly propagate from the torso through the neck. Our simulation results indicated that such a blood surge reaches the base of the brain and induces considerably high wall shear stresses as it propagates through the cerebrovascular network, supporting the plausibility of vascular-level injury due to the indirect mechanism. In contrast, because the predicted brain-tissue strains are much lower than the thresholds for tissue-level damage identified in literature, our results suggest that strain-induced damage to the brain tissue solely due to the indirect mechanism is unlikely.

## Data Availability Statement

The datasets presented in this article are not readily available because a written request to the corresponding author along with a summary of the planned research are required to obtain the datasets and related analyses. Requests to access the datasets should be directed to jaques.reifman.civ@mail.mil.

## Ethics Statement

The animal study was reviewed and approved by The Institutional Animal Care and Use Committees at the New Jersey Institute of Technology (Newark, NJ, United States) and The University of Utah (Salt Lake City, UT, United States), as well as the Animal Care and Use Review Office of the United States Army Medical Research and Development Command (Ft. Detrick, MD, United States).

## Author Contributions

GU, KM, NC, and JR designed the study. JER developed the computational models, performed the simulations, and analyzed the results. JER and JR drafted the manuscript. MS, EA, and NC performed the experiments and analyzed the experimental data. KM and SY conducted the imaging procedure. AS, DS, VK, GU, and JR contributed to the evaluation of the experimental and simulated data. AS, DS, VK, and GU contributed to the preparation of the manuscript. All authors have reviewed the manuscript and approved the submitted version.

## Disclaimer

The opinions or assertions contained herein are the private views of the authors and are not to be construed as official or reflecting the views of the United States (U.S.) Army, the Department of Defense (DoD), or The Henry M. Jackson Foundation for the Advancement of Military Medicine, Inc. (HJF). Any citations of commercial organizations and trade names in this report do not constitute an official U.S. Army, DoD, or HJF endorsement of approval of the products or services of these organizations. This paper has been approved for public release with unlimited distribution.

## Conflict of Interest

The authors declare that the research was conducted in the absence of any commercial or financial relationships that could be construed as a potential conflict of interest.

## References

[B1] Alay E. Skotak M. Misistia A. Chandra N. (2018). Dynamic loads on human and animal surrogates at different test locations in compressed-gas-driven shock tubes. *Shock Waves* 28 51–62. 10.1007/s00193-017-0762-4

[B2] Ambrosi D. Quarteroni A. Rozza G. (2012). *Modeling of Physiological Flows.* Milan: Springer.

[B3] Aoki T. Nishimura M. Matsuoka T. Yamamoto K. Furuyashiki T. Kataoka H. (2011). PGE(2) -EP(2) signalling in endothelium is activated by haemodynamic stress and induces cerebral aneurysm through an amplifying loop via NF-κB. *Br. J. Pharmacol.* 163 1237–1249. 10.1111/j.1476-5381.2011.01358.x 21426319 PMC3144537

[B4] Assari S. Laksari K. Barbe M. Darvish K. (2013). “Cerebral blood pressure rise during blast exposure in a rat model of blast-induced traumatic brain injury,” in *Proceedings of the ASME 2013 International Mechanical Engineering Congress and Exposition*, (V03AT03A016) (San Diego, CA).

[B5] Bain A. C. Meaney D. F. (2000). Tissue-level thresholds for axonal damage in an experimental model of central nervous system white matter injury. *J. Biomech. Eng.* 122 615–622. 10.1115/1.1324667 11192383

[B6] Bell E. D. Converse M. Mao H. Unnikrishnan G. Reifman J. Monson K. L. (2018). Material properties of rat middle cerebral arteries at high strain rates. *J. Biomech. Eng.* 140:071004 10.1115/1.4039625 29560495

[B7] Bolander R. Mathie B. Bir C. Ritzel D. VandeVord P. (2011). Skull flexure as a contributing factor in the mechanism of injury in the rat when exposed to a shock wave. *Ann. Biomed. Eng.* 39 2550–2559. 10.1007/s10439-011-0343-0 21735320

[B8] Bragin D. E. Bush R. C. Nemoto E. M. (2013). Effect of cerebral perfusion pressure on cerebral cortical microvascular shunting at high intracranial pressure in rats. *Stroke* 44 177–181. 10.1161/strokeaha.112.668293 23204051 PMC3586667

[B9] Cater H. L. Sundstrom L. E. Morrison B. III (2006). Temporal development of hippocampal cell death is dependent on tissue strain but not strain rate. *J. Biomech.* 39 2810–2818. 10.1016/j.jbiomech.2005.09.023 16289515

[B10] Cernak I. (2010). The importance of systemic response in the pathobiology of blast-induced neurotrauma. *Front. Neurol.* 1:151. 10.3389/fneur.2010.00151 21206523 PMC3009449

[B11] Cernak I. (2015). “Blast injuries and blast-induced neurotrauma: overview of pathophysiology and experimental knowledge models and findings,” in *Brain Neurotrauma: Molecular, Neuropsychological, and Rehabilitation Aspects*, ed. Kobeissy F. H. (Boca Raton, FL: CRC Press). 26269895

[B12] Chalouhi N. Ali M. S. Jabbour P. M. Tjoumakaris S. I. Gonzalez L. F. Rosenwasser R. H. (2012). Biology of intracranial aneurysms: role of inflammation. *J. Cereb. Blood Flow Metab.* 32 1659–1676. 10.1038/jcbfm.2012.84 22781330 PMC3434628

[B13] Chavko M. Watanabe T. Adeeb S. Lankasky J. Ahlers S. T. McCarron R. M. (2011). Relationship between orientation to a blast and pressure wave propagation inside the rat brain. *J. Neurosci. Methods* 195 61–66. 10.1016/j.jneumeth.2010.11.019 21129403

[B14] Clemedson C. J. (1956). Shock wave transmission to the central nervous system. *Acta Physiol. Scand.* 37 204–214. 10.1111/j.1748-1716.1956.tb01356.x 13361900

[B15] Clemedson C. J. Criborn C. O. (1955). Mechanical response of different parts of a living body to a high explosive shock wave impact. *Am. J. Physiol.* 181 471–476. 10.1152/ajplegacy.1955.181.3.471 13238584

[B16] Clemedson C. J. Pettersson H. (1956). Propagation of a high explosive air shock wave through different parts of an animal body. *Am. J. Physiol.* 184 119–126. 10.1152/ajplegacy.1955.184.1.119 13283101

[B17] Cosson E. Herisse M. Laude D. Thomas F. Valensi P. Attali J. R. (2007). Aortic stiffness and pulse pressure amplification in Wistar-Kyoto and spontaneously hypertensive rats. *Am. J. Physiol. Heart Circ. Physiol.* 292 H2506–H2512. 10.1152/ajpheart.00732.2006 17237248

[B18] Fievisohn E. Bailey Z. Guettler A. VandeVord P. (2018). Primary blast brain injury mechanisms: current knowledge, limitations, and future directions. *J. Biomech. Eng.* 140:020806 10.1115/1.4038710 29222564

[B19] Gama Sosa M. A. De Gasperi R. Janssen P. L. Yuk F. J. Anazodo P. C. Pricop P. E. (2014). Selective vulnerability of the cerebral vasculature to blast injury in a rat model of mild traumatic brain injury. *Acta Neuropathol. Commun.* 2:67. 10.1186/2051-5960-2-67 24938728 PMC4229875

[B20] Gama Sosa M. A. De Gasperi R. Paulino A. J. Pricop P. E. Shaughness M. C. Maudlin-Jeronimo E. (2013). Blast overpressure induces shear-related injuries in the brain of rats exposed to a mild traumatic brain injury. *Acta Neuropathol. Commun.* 1:51. 10.1186/2051-5960-1-51 24252601 PMC3893550

[B21] Gama Sosa M. A. De Gasperi R. Perez Garcia G. S. Perez G. M. Searcy C. Vargas D. (2019). Low-level blast exposure disrupts gliovascular and neurovascular connections and induces a chronic vascular pathology in rat brain. *Acta Neuropathol. Commun.* 7:6. 10.1186/s40478-018-0647-5 30626447 PMC6327415

[B22] Geddes D. M. Cargill R. S. II LaPlaca M. C. (2003). Mechanical stretch to neurons results in a strain rate and magnitude-dependent increase in plasma membrane permeability. *J. Neurotrauma* 20 1039–1049. 10.1089/089771503770195885 14588120

[B23] Goeller J. Wardlaw A. Treichler D. O’Bruba J. Weiss G. (2012). Investigation of cavitation as a possible damage mechanism in blast-induced traumatic brain injury. *J. Neurotrauma* 29 1970–1981. 10.1089/neu.2011.2224 22489674

[B24] Goldstein L. E. Fisher A. M. Tagge C. A. Zhang X. L. Velisek L. Sullivan J. A. (2012). Chronic traumatic encephalopathy in blast-exposed military veterans and a blast neurotrauma mouse model. *Sci. Transl. Med.* 4:134ra160. 10.1126/scitranslmed.3003716 22593173 PMC3739428

[B25] Harper S. L. Bohlen H. G. (1984). Microvascular adaptation in the cerebral cortex of adult spontaneously hypertensive rats. *Hypertension* 6 408–419. 10.1161/01.HYP.6.3.408 6735460

[B26] Haslach H. W. Jr. Gipple J. M. Leahy L. N. (2017). Influence of high deformation rate, brain region, transverse compression, and specimen size on rat brain shear stress morphology and magnitude. *J. Mech. Behav. Biomed. Mater.* 68 88–102. 10.1016/j.jmbbm.2017.01.036 28157598

[B27] Heyburn L. Abutarboush R. Goodrich S. Urioste R. Batuure A. Statz J. (2019). Repeated low-level blast overpressure leads to endovascular disruption and alterations in TDP-43 and Piezo2 in a rat model of blast TBI. *Front. Neurol.* 10:766. 10.3389/fneur.2019.00766 31417481 PMC6682625

[B28] Koliatsos V. E. Cernak I. Xu L. Song Y. Savonenko A. Crain B. J. (2011). A mouse model of blast injury to brain: initial pathological, neuropathological, and behavioral characterization. *J. Neuropathol. Exp. Neurol.* 70 399–416. 10.1097/NEN.0b013e3182189f06 21487304

[B29] Kuriakose M. Rama Rao K. V. Younger D. Chandra N. (2018). Temporal and spatial effects of blast overpressure on blood-brain barrier permeability in traumatic brain injury. *Sci. Rep.* 8:8681. 10.1038/s41598-018-26813-7 29875451 PMC5989233

[B30] Kuriakose M. Skotak M. Misistia A. Kahali S. Sundaramurthy A. Chandra N. (2016). Tailoring the blast exposure conditions in the shock tube for generating pure, primary shock waves: the end plate facilitates elimination of secondary loading of the specimen. *PLoS One* 11:e0161597. 10.1371/journal.pone.0161597 27603017 PMC5014318

[B31] Kuriakose M. Younger D. Ravula A. R. Alay E. Rama Rao K. V. Chandra N. (2019). Synergistic role of oxidative stress and blood-brain barrier permeability as injury mechanisms in the acute pathophysiology of blast-induced neurotrauma. *Sci. Rep.* 9:7717. 10.1038/s41598-019-44147-w 31118451 PMC6531444

[B32] Lehoux S. Castier Y. Tedgui A. (2006). Molecular mechanisms of the vascular responses to haemodynamic forces. *J. Intern. Med.* 259 381–392. 10.1111/j.1365-2796.2006.01624.x 16594906

[B33] Leonardi A. D. Bir C. A. Ritzel D. V. VandeVord P. J. (2011). Intracranial pressure increases during exposure to a shock wave. *J. Neurotrauma* 28 85–94. 10.1089/neu.2010.1324 21091267

[B34] Liu Y. Dang C. Garcia M. Gregersen H. Kassab G. S. (2007). Surrounding tissues affect the passive mechanics of the vessel wall: theory and experiment. *Am. J. Physiol. Heart Circ. Physiol.* 293 H3290–H3300. 10.1152/ajpheart.00666.2007 17873018

[B35] Logsdon A. F. Meabon J. S. Cline M. M. Bullock K. M. Raskind M. A. Peskind E. R. (2018). Blast exposure elicits blood-brain barrier disruption and repair mediated by tight junction integrity and nitric oxide dependent processes. *Sci. Rep.* 8:11344. 10.1038/s41598-018-29341-6 30054495 PMC6063850

[B36] Lu D. Kassab G. S. (2011). Role of shear stress and stretch in vascular mechanobiology. *J. R. Soc. Interface* 8 1379–1385. 10.1098/rsif.2011.0177 21733876 PMC3163429

[B37] Maier G. W. Tyson G. S. Jr. Olsen C. O. Kernstein K. H. Davis J. W. Conn E. H. (1984). The physiology of external cardiac massage: high-impulse cardiopulmonary resuscitation. *Circulation* 70 86–101. 10.1161/01.cir.70.1.86 6723014

[B38] Needham C. E. Ritzel D. Rule G. T. Wiri S. Young L. (2015). Blast testing issues and TBI: experimental models that lead to wrong conclusions. *Front. Neurol.* 6:72. 10.3389/fneur.2015.00072 25904891 PMC4389725

[B39] Papaioannou T. G. Stefanadis C. (2005). Vascular wall shear stress: basic principles and methods. *Hellenic J. Cardiol.* 46 9–15. 15807389

[B40] Rodriguez U. A. Zeng Y. Deyo D. Parsley M. A. Hawkins B. E. Prough D. S. (2018). Effects of mild blast traumatic brain injury on cerebral vascular, histopathological, and behavioral outcomes in rats. *J. Neurotrauma* 35 375–392. 10.1089/neu.2017.5256 29160141 PMC5784797

[B41] Rudikoff M. T. Maughan W. L. Effron M. Freund P. Weisfeldt M. L. (1980). Mechanisms of blood flow during cardiopulmonary resuscitation. *Circulation* 61 345–352. 10.1161/01.CIR.61.2.345 7351060

[B42] Salzar R. S. Treichler D. Wardlaw A. Weiss G. Goeller J. (2017). Experimental investigation of cavitation as a possible damage mechanism in blast-induced traumatic brain injury in post-mortem human subject heads. *J. Neurotrauma* 34 1589–1602. 10.1089/neu.2016.4600 27855566

[B43] Schierling W. Troidl K. Mueller C. Troidl C. Wustrack H. Bachmann G. (2009). Increased intravascular flow rate triggers cerebral arteriogenesis. *J. Cereb. Blood Flow Metab.* 29 726–737. 10.1038/jcbfm.2008.165 19142189

[B44] Simard J. M. Pampori A. Keledjian K. Tosun C. Schwartzbauer G. Ivanova S. (2014). Exposure of the thorax to a sublethal blast wave causes a hydrodynamic pulse that leads to perivenular inflammation in the brain. *J. Neurotrauma* 31 1292–1304. 10.1089/neu.2013.3016 24673157 PMC4108981

[B45] Sriram K. Intaglietta M. Tartakovsky D. M. (2014). Non-newtonian flow of blood in arterioles: consequences for wall shear stress measurements. *Microcirculation* 21 628–639. 10.1111/micc.12141 24703006 PMC4185264

[B46] Sundaramurthy A. Alai A. Ganpule S. Holmberg A. Plougonven E. Chandra N. (2012). Blast-induced biomechanical loading of the rat: an experimental and anatomically accurate computational blast injury model. *J. Neurotrauma* 29 2352–2364. 10.1089/neu.2012.2413 22620716

[B47] Suneson A. Hansson H. A. Seeman T. (1990a). Pressure wave injuries to the nervous system caused by high-energy missile extremity impact: Part i. Local and distant effects on the peripheral nervous system–a light and electron microscopic study on pigs. *J. Trauma* 30 281–294. 10.1097/00005373-199003000-00006 2313747

[B48] Suneson A. Hansson H. A. Seeman T. (1990b). Pressure wave injuries to the nervous system caused by high-energy missile extremity impact: Part ii. Distant effects on the central nervous system–a light and electron microscopic study on pigs. *J. Trauma* 30 295–306. 10.1097/00005373-199003000-00007 2313748

[B49] Szymanski M. P. Metaxa E. Meng H. Kolega J. (2008). Endothelial cell layer subjected to impinging flow mimicking the apex of an arterial bifurcation. *Ann. Biomed. Eng.* 36 1681–1689. 10.1007/s10439-008-9540-x 18654851 PMC2570750

[B50] Taylor P. A. Ford C. C. (2009). Simulation of blast-induced early-time intracranial wave physics leading to traumatic brain injury. *J. Biomech. Eng.* 131:061007 10.1115/1.3118765 19449961

[B51] Unnikrishnan G. Mao H. Sundaramurthy A. Bell E. D. Yeoh S. Monson K. (2019). A 3-D rat brain model for blast-wave exposure: effects of brain vasculature and material properties. *Ann. Biomed. Eng.* 47 2033–2044. 10.1007/s10439-019-02277-2 31054004 PMC6757019

[B52] Vovenko E. (1999). Distribution of oxygen tension on the surface of arterioles, capillaries and venules of brain cortex and in tissue in normoxia: An experimental study on rats. *Pflugers Arch.* 437 617–623. 10.1007/s004240050825 10089576

[B53] Vrselja Z. Brkic H. Mrdenovic S. Radic R. Curic G. (2014). Function of circle of Willis. *J. Cereb. Blood Flow Metab.* 34 578–584. 10.1038/jcbfm.2014.7 24473483 PMC3982101

[B54] Wang S. H. Lee L. P. Lee J. S. (2001). A linear relation between the compressibility and density of blood. *J. Acoust. Soc. Am.* 109 390–396. 10.1121/1.1333419 11206168

[B55] Weisfeldt M. L. Chandra N. Tsitlik J. (1981). Increased intrathoracic pressure—not direct heart compression—causes the rise in intrathoracic vascular pressures during CPR in dogs and pigs. *Crit. Care Med.* 9 377–378. 10.1097/00003246-198105000-00012 7214961

[B56] Zhang L. Yang K. H. King A. I. (2004). A proposed injury threshold for mild traumatic brain injury. *J. Biomech. Eng.* 126 226–236. 10.1115/1.1691446 15179853

