# Supplementary figures and images for: Does Blast Exposure to the Torso Cause a Blood Surge to the Brain?
Source: Front Bioeng Biotechnol. 2020 Dec 17;8:573647. doi: 10.3389/fbioe.2020.573647 (PMC7773947; doi:10.3389/fbioe.2020.573647)

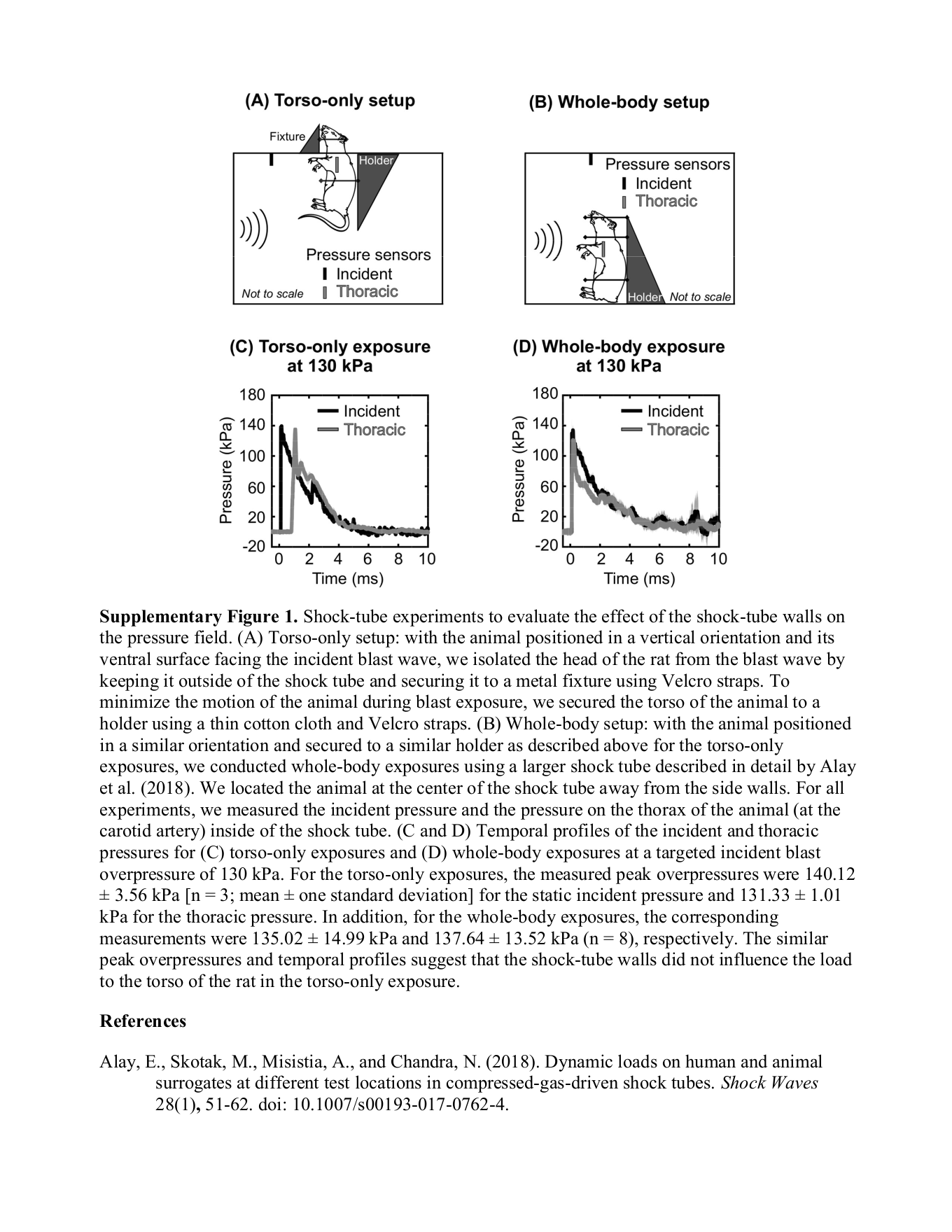

Supplement: Supplementary file 1 [file Image_1.tiff]

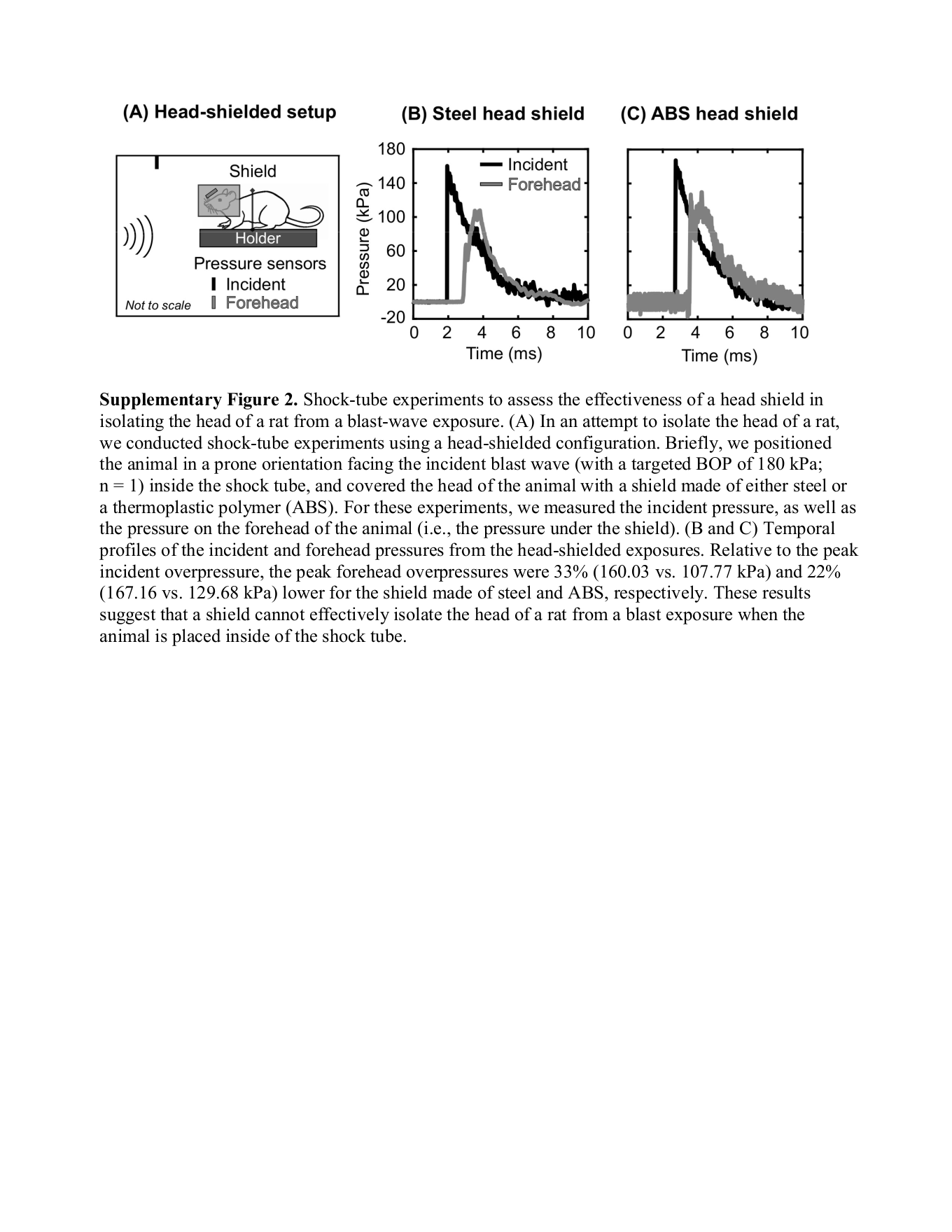

Supplement: Supplementary file 2 [file Image_2.tiff]

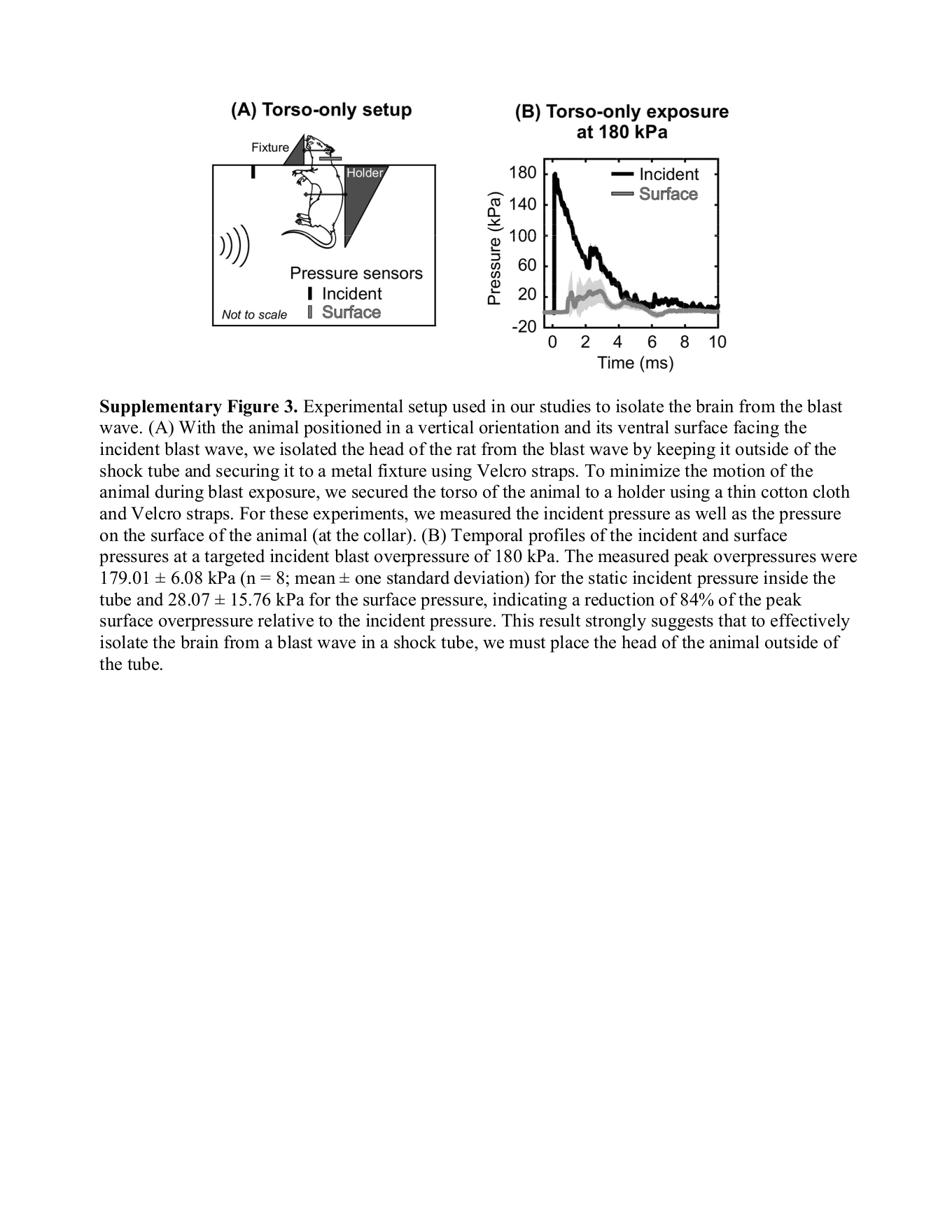

Supplement: Supplementary file 3 [file Image_3.tiff]
